# Elevated E200K Somatic Mutation of the Prion Protein Gene (*PRNP*) in the Brain Tissues of Patients with Sporadic Creutzfeldt–Jakob Disease (CJD)

**DOI:** 10.3390/ijms241914831

**Published:** 2023-10-02

**Authors:** Sae-Young Won, Yong-Chan Kim, Byung-Hoon Jeong

**Affiliations:** 1 Korea Zoonosis Research Institute, Jeonbuk National University, 820-120, Hana-ro, Iksan 54531, Republic of Korea; gkfh32@jbnu.ac.kr; 2 Department of Bioactive Material Sciences, Institute for Molecular Biology and Genetics, Jeonbuk National University, Jeonju 54896, Republic of Korea; 3 Department of Biological Sciences, Andong National University, Andong 36729, Republic of Korea; kych@anu.ac.kr

**Keywords:** prion, sporadic CJD, somatic mutation, pyrosequencing, E200K, V203I

## Abstract

Sporadic Creutzfeldt–Jakob disease (CJD) is a major human prion disease worldwide. CJD is a fatal neurodegenerative disease caused by an abnormal prion protein (PrP^Sc^). To date, the exact etiology of sporadic CJD has not been fully elucidated. We investigated the E200K and V203I somatic mutations of the prion protein gene (*PRNP*) in sporadic CJD patients and matched healthy controls using pyrosequencing. In addition, we estimated the impact of somatic mutations on the human prion protein (PrP) using PolyPhen-2, PANTHER and PROVEAN. Furthermore, we evaluated the 3D structure and electrostatic potential of the human PrP according to somatic mutations using DeepView. The rates of *PRNP* K200 somatic mutation were significantly increased in the frontal cortex and hippocampus of sporadic CJD patients compared to the matched controls. In addition, the electrostatic potential of the human PrP was significantly changed by the K200 somatic mutation of the *PRNP* gene. To the best of our knowledge, this is the first report on an association of the *PRNP* K200 somatic mutation with sporadic CJD.

## 1. Introduction

Prion diseases are rapidly progressive and fatal neurodegenerative disorders caused by the conversion of a normal prion protein (PrP^C^) to an abnormal prion protein (PrP^Sc^) [1,2,3,4,5,6,7]. In humans, the representative form of prion disease is Creutzfeldt–Jakob disease (CJD), which is divided into four subtypes: iatrogenic, genetic, variant and sporadic CJD [8,9,10,11]. Of these four CJDs, the causes of iatrogenic, variant and genetic CJD have been identified. Variant CJD is caused by the consumption of PrP^Sc^-contaminated bovine meat. In addition, iatrogenic CJD is transmitted by blood transfusions and the cross-contamination of surgical instruments, accounting for less than 1% of all cases [12,13]. Genetic CJD is caused by pathogenic germline mutations in the prion protein gene (*PRNP)* [14]. Sporadic CJD accounts for approximately 85% of all human prion diseases and occurs worldwide in 1–2 cases per million people per year [15]. However, the exact etiology of the most frequently observed form of CJD, sporadic CJD, has not been fully elucidated thus far. Therefore, investigation of the cause of sporadic CJD is needed.

Recent studies have reported that somatic mutations in the brain cause the onset of several neurodegenerative diseases, including Alzheimer’s disease, Parkinson’s disease and CJD [16,17,18,19,20]. In Alzheimer’s disease, somatic mutations in the presenilin-1 (*PSEN1*) gene have been reported in peripheral lymphocytes (8%) and in the cerebral cortex (14%) derived from a patient with Alzheimer’s disease [21]. Another study reported nine somatic mutations in the amyloid beta precursor protein (*APP*), sortilin-related receptor 1 (*SORL1*), nicastrin (*NCSTN*) and microtubule affinity-regulating kinase 4 (*MARK4*) genes in patients with Alzheimer’s disease by next-generation sequencing (NGS) [22]. In Parkinson’s disease, an average of three somatic mutations of the genes related to synaptic functions have been found in the brain derived from patients with Parkinson’s disease by whole exome sequencing (WES) [19]. In a sporadic CJD patient, a somatic mutation of the *PRNP* gene was detected in peripheral blood cells and brain tissue using Sanger sequencing [18]. However, since somatic mutations can be observed even in the human brain with normal aging, there remains a question of whether the detected somatic mutations have a significant value.

In this study, we collected hippocampal and frontal cortex brain samples from sporadic CJD patients and age- and sex-matched controls. In addition, we purified genomic DNA from brain samples and performed pyrosequencing to identify the E200K and V203I somatic mutations in the human *PRNP* gene, which are frequently reported mutations in familial CJD patients. Furthermore, we estimated the impact of E200K and V203I somatic mutations on the human prion protein (PrP) in silico using the programs PolyPhen-2, PANTHER and PROVEAN [23,24,25].

## 2. Results

### 2.1. Investigation of Somatic Mutations of the Human PRNP Gene by Pyrosequencing

**Figure 1:**
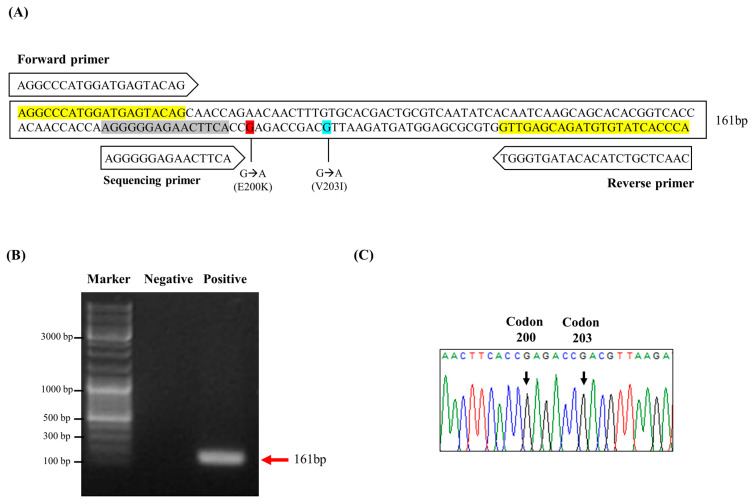
The first pyrosequencing scheme to detect somatic mutations of the human prion protein gene (*PRNP*). (**A**) Schematic design of primers for pyrosequencing to detect E200K and V203I somatic mutations of the human *PRNP* gene. (**B**) Gene-specific primers were used to obtain 161 bp amplicons of the human *PRNP* gene containing codons 200 and 203. Negative: PCR product with sterilized distilled water instead of the template. Positive: PCR product with genomic DNA extracted from a normal aging human brain. (**C**) Representative electropherograms of pyrosequencing containing codons 200 and 203 of the *PRNP* gene results. CTL: normal aging control. CJD: Creutzfeldt–Jakob disease patient.

To detect somatic mutations of E200K and V203I in the *PRNP* gene in human brain samples, pyrosequencing analysis was performed. Detailed information on the detection primers for pyrosequencing is presented in Figure 1A.

**Figure 2:**
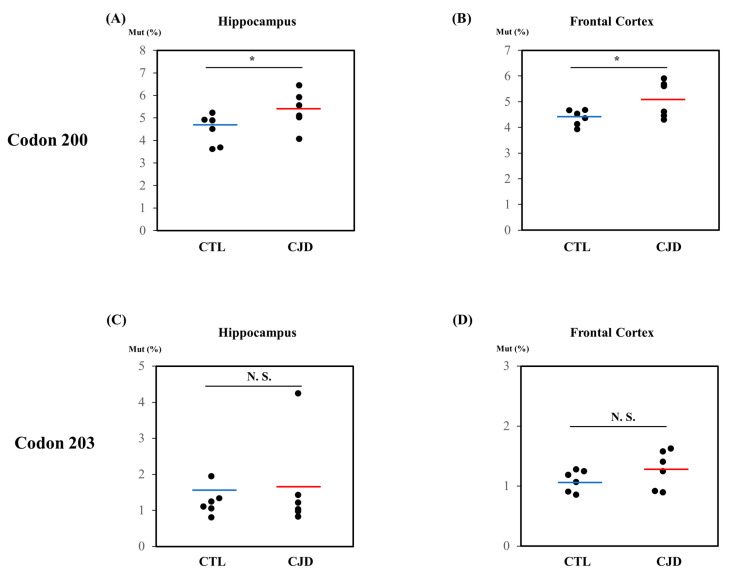
Comparison of somatic mutation rates of the *PRNP* gene in brains from sporadic CJD patients and matched controls. (**A**) Comparison of K200 somatic mutation rates of the *PRNP* gene in the hippocampus. (**B**) Comparison of K200 somatic mutation rates of the *PRNP* gene in the frontal cortex. (**C**) Comparison of I203 somatic mutation rates of the *PRNP* gene in the hippocampus. (**D**) Comparison of I203 somatic mutation rates of the *PRNP* gene in the frontal cortex. *: *p* < 0.05; N.S.: not significant. Red and blue horizontal bars indicate the average somatic mutation rates of the *PRNP* gene in brains from sporadic CJD patients and matched controls, respectively.

The pyrosequencing primers targeting K200 and I203 of the human *PRNP* gene were designed using the PyroMark Assay Design program (QIAGEN, Hilden, Germany). Biotinylated PCR products were amplified by PCR and the length was confirmed on a gel (161 bp) (Figure 1B). DNA peaks at codons 200 and 203 of the *PRNP* gene were successfully detected by pyrosequencing (Figure 1C). In addition, we performed a pyrosequencing analysis in the hippocampus and frontal cortex derived from six sporadic CJD patients and six matched controls. Importantly, the rates of *PRNP* K200 somatic mutation in the hippocampus and frontal cortex of the sporadic CJD patients were higher than those of the matched controls. (*p* < 0.05). However, the rates of *PRNP* I203 somatic mutation were not significantly different in the hippocampus and frontal cortex between the sporadic CJD patients and the controls (Figure 2).

### 2.2. Evaluation of the Effect of Somatic Mutations on the Human PrP Using in Silico Analysis

To evaluate the impact of E200K and V203I mutations on human PrP, we utilized PolyPhen-2, PANTHER and PROVEAN. The E200K and V203I mutations by PolyPhen-2 were predicted to be ‘Probably damaging’ with a score of 0.987 and to be ‘Possibly damaging’ with a score of 0.882, respectively. The E200K and V203I mutations by PANTHER were estimated to be ‘Possibly damaging’ with a score of 361 and to be ‘Probably benign’ with a score of 176, respectively. The E200K and V203I mutations by PROVEAN were evaluated to be ‘Neutral’ with a score of −1.478 and to be ‘Neutral’ with a score of −0.004, respectively (Table 1).

### 2.3. Evaluation of the Effect of Somatic Mutations on the Electrostatic Potential of the PrP

**Figure 3:**
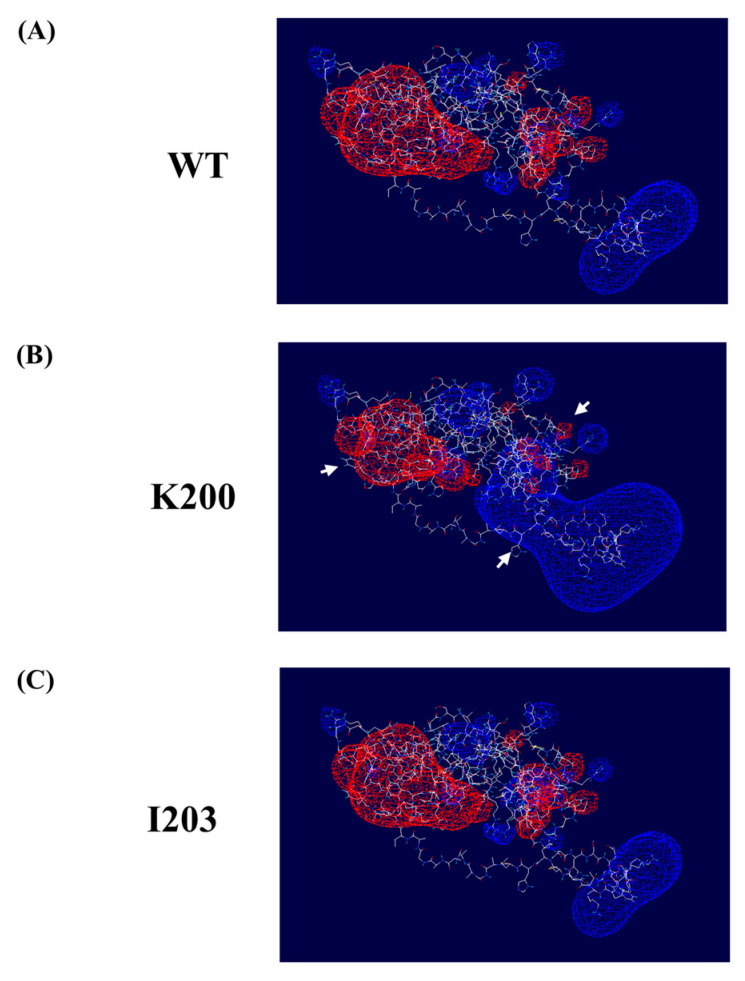
The three-dimensional (3D) structure and electrostatic potential of the human prion protein (PrP) according to somatic mutations in the *PRNP* gene. (**A**) The 3D structure and electrostatic potential of the wild-type human PrP. (**B**) The 3D structure and electrostatic potential of the human PrP with the K200 allele. (**C**) The 3D structure and electrostatic potential of the human PrP with the I203 allele. Positive potentials are drawn in blue. Negative potentials are drawn in red. White arrows indicate a significant change in electrostatic potential compared to the wild-type PrP.

We evaluated the electrostatic potential and 3D structure analyses of the human PrP according to the somatic mutations of the *PRNP* gene (Figure 3). In a human PrP with the K200 allele, an increase in the positive charge and a decrease in the negative charge were observed adjacent to codon 200 compared to a wild-type human PrP (Figure 3A,B). However, we did not find significant changes in the electrostatic potential and 3D structure of the human PrP based on the I203 mutation (Figure 3).

## 3. Discussion

To date, the mechanism responsible for the conversion of a PrP^C^ into a PrP^Sc^ in sporadic CJD has not been identified. Previous studies have reported that aging increases somatic mutations in neurons, and the accumulated mutations of the disease-associated genes encoding DNA repair cause several neurodegenerative diseases [26]. Although several studies have investigated somatic mutations of the *PRNP* gene in sporadic CJD patients, studies using age-matched controls have not been reported thus far. Since somatic mutations occur even in the normal aging process, a comparison is necessary to determine whether the somatic mutation rates observed in sporadic CJD are unusually high.

In the present study, higher K200 somatic mutations of the *PRNP* gene were observed in sporadic CJD patients than in age- and sex-matched controls (Figure 2). We also found that the K200 mutation of the *PRNP* gene significantly changed the electrostatic potential of the human PrP (Figure 3). Recent studies have reported that the K200 mutation of the *PRNP* gene has shown high penetrance and was confirmed as a causal factor of prion diseases using a transgenic mouse model. Since the causal relationship of K200 somatic mutation has been demonstrated in previous studies, the elevation of K200 somatic mutation rates found in this study may be an important factor in the occurrence of sporadic CJD. However, this investigation was carried out with a relatively small sample size. In addition, E200K is known to be highly prevalent in certain European countries, accounting for the majority of prion disease cases [14], and there is a possibility of misdiagnosed familial CJD in the samples with elevated E200K in the present study. Thus, further validation in a larger sample size is an important future step.

In the current study, we investigated only two somatic mutations of the *PRNP* gene. However, previous studies have reported that over 40 germline mutations were related to several genetic prion diseases, including CJD, Gerstmann–Sträussler–Scheinker syndrome (GSS) and fatal familial insomnia (FFI) [8,14,27,28]. Thus, further investigation of other somatic mutations in the *PRNP* gene is needed to confirm whether the elevated K200 somatic mutations of the *PRNP* gene are the only functionally relevant mutation of sporadic CJD.

We did not find any differences in the I203 somatic mutation rates of the *PRNP* gene between sporadic CJD patients and matched controls (Figure 2). The V203I mutation of the *PRNP* gene has been reported in several case reports of genetic CJD in Italy, Korea, China and Japan. However, a recent systemic review concluded that the I203 mutation of the *PRNP* gene did not exhibit high penetrance and could be benign or is only associated with susceptibility to prion disease. We also found that the I203 mutation of the *PRNP* gene did not affect significant changes in the electrostatic potential of the human PrP (Figure 3). These studies indicate that I203 mutations of the *PRNP* gene may not be a crucial factor in sporadic CJD.

Herein, we identified elevated K200 somatic mutations in the *PRNP* gene in the frontal cortex and hippocampus of sporadic CJD patients. In addition, unlike the I203 mutation of the *PRNP* gene, the K200 mutation induced a significant change in the electrostatic potential of the human PrP. To the best of our knowledge, this is the first report of the association of the K200 somatic mutation of the *PRNP* gene with sporadic CJD patients.

## 4. Materials and Methods

### 4.1. Subjects

The 24 brain samples, including frontal cortex and hippocampus, of 6 sporadic CJD patients and 6 age- and sex-matched controls were provided by the University of Edinburgh in the United Kingdom. Sporadic CJD was diagnosed by an autopsy according to the guidelines of the World Health Organization (WHO). Detailed information on the samples is presented in Table 2.

### 4.2. Ethics Statements

All experimental procedures were approved by the institutional review board (IRB) of Jeonbuk National University and in accordance with the 1964 Declaration of Helsinki and its later amendments or comparable ethical standards (approval number: 2020-10-014). All the samples and related information were anonymized prior to research.

### 4.3. Genomic DNA Extraction

Genomic DNA was isolated from 20 mg of brain tissue using the Tissue Genomic DNA Isolation Kit (QIAGEN, Germantown, MD, USA) following the manufacturer’s instructions. The quality and concentration of genomic DNA (15–20 ng/µL, A260/280 = 1.8–2.0) were evaluated using a Nanodrop One spectrophotometer (Thermo Scientific, Waltham, MA, USA).

### 4.4. Polymerase Chain Reaction (PCR)

PCR was performed to amplify fragments containing the E200K and V203I SNPs of the human *PRNP* gene with Pfu DNA Polymerase (BIOFACT, Daejeon, Korea). The experimental conditions of each primer for PCR are described in Table 3. The PCR mixture contained 10 pmol of each primer, 2.5 µL of 10 × Taq DNA polymerase buffer, 1 μL of 10 mM dNTP mixture and 2.5 units of Taq DNA polymerase.

### 4.5. Sanger Sequencing

The purification of the PCR products was performed using a PCR Purification Kit (Thermo Fisher Scientific, USA), and the sequence was analyzed using an ABI 3730 automatic sequencer (ABI, Foster City, CA, USA). Sequencing results were analyzed by using Finch TV software (Geospiza Inc., Denver, CO, USA).

### 4.6. Pyrosequencing

The biotinylated PCR products of the human *PRNP* gene were amplified by nonbiotinylated forward and biotinylated reverse primers. The volume of the total PCR mixture was 80 µL, including 2 µL Streptoavidin Sepharose High Performance Medium (GE Healthcare, Chicago, IL, USA), 40 µL PyroMark Binding Buffer (QIAGEN, USA), 8.5 µL high purity autoclaved water and 10 µL PCR products, and shaken for 15 min at 14,000 rpm. The PCR products were washed sequentially with 70% ethanol for 10 sec, PyroMark Denaturation Buffer (QIAGEN, USA) for 10 sec and PyroMark Wash Buffer (QIAGEN, USA) for 10 sec and then mixed with 24.2 µL Pyromark annealing Buffer (QIAGEN, USA) and 0.8 µL sequencing primer (2 µM). Finally, the samples were incubated in a PyroMark Q25 plate holder preheated to 80 °C for 2 min. Prepared samples were loaded into PyroMark Q24 (QIAGEN, USA) and operated in allele quantification (AQ) mode according to the manufacturer’s protocol.

### 4.7. In Silico Estimation of the Impact of E200K and V203I Somatic Mutations on the Human Prion Protein (PrP)

The biological impacts of E200K and V203I somatic mutations on the human PrP were evaluated by using the PolyPhen-2 (http://genetics.bwh.harvard.edu/pph2/index.shtml (accessed on 14 April 2022 )), PANTHER (http://www.pantherdb.org/ (accessed on 14 April 2022)) and PROVEAN (http://provean.jcvi.org/seq_submit.php (accessed on 14 April 2022)) programs. PolyPhen-2 predicts the effect on the stability and function of amino acid changes using an independent count (PSIC). PANTHER calculates the length of time (in millions of years) of a position in a protein sequence using a hidden Markov model (HMM) based on statistical modeling. PROVEAN is a program that predicts the impact of nonsynonymous SNPs and indels on protein function.

### 4.8. D Structure and Electrostatic Potential Analyses

The NMR structure of the human PrP was obtained from the RCSB Protein Data Bank (PDB ID: 6FNV.1.A). The 3D structure of the human PrP was analyzed by using the SWISS-MODEL program (https://swissmodel.expasy.org/ (accessed on 14 April 2022)). The electrostatic potential according to the somatic mutations of the human *PRNP* gene was analyzed by using the Swiss-PdbViewer 4.1 program (https://spdbv.vital-it.ch/ (accessed on 14 April 2022)).

### 4.9. Statistical Analysis

Statistical analyses were performed by using SAS version 9.4 (SAS Institute Inc., Cary, NC, USA). The symbol * indicates *p* < 0.05. ‘N.S.’ indicates not significant.

## Figures and Tables

**Table 1:** In silico evaluation of the impact of K200 and I203 somatic mutations on the human prion protein (PrP).

Somatic Mutations	Methods	Score	Prediction
c.598G > A (E200K)	PolyPhen-2	0.987	Probably damaging
	PANTHER	361	Possibly damaging
	PROVEAN	−1.478	Neutral
c.607G > A (V203I)	PolyPhen-2	0.882	Possibly damaging
	PANTHER	176	Probably benign
	PROVEAN	−0.004	Neutral

**Table 2:** K200 and I203 somatic mutation rates of the prion protein gene (*PRNP*) observed in sporadic CJD patients and matched controls.

Sample No.	Type	Sex	Age	Average	Brain Tissue	K200 Somatic Mutation Rates (%)	I203 Somatic Mutation Rates (%)	Codon 129 Genotype
Sample 1	Control 1	Male	84	64.6 ± 9.2	Frontal cortex	4.13	1.19	MM
Sample 2					Hippocampus	4.92	1.25	
Sample 3	Control 2	Male	67		Frontal cortex	4.68	1.25	MM
Sample 4					Hippocampus	4.51	0.81	
Sample 5	Control 3	Male	73		Frontal cortex	4.67	1.28	VV
Sample 6					Hippocampus	5.23	1.95	
Sample 7	Control 4	Female	57		Frontal cortex	4.36	0.91	MV
Sample 8					Hippocampus	3.62	1.06	
Sample 9	Control 5	Female	73		Frontal cortex	3.93	0.86	MV
Sample 10					Hippocampus	3.69	1.11	
Sample 11	Control 6	Female	53		Frontal cortex	4.53	1.07	MV
Sample 12					Hippocampus	4.89	1.34	
Sample 13	Case 1	Male	86	70.7 ± 13.3	Frontal cortex	5.68	0.92	MM
Sample 14					Hippocampus	4.07	4.25	
Sample 15	Case 2	Male	66		Frontal cortex	4.61	1.41	MV
Sample 16					Hippocampus	5.03	0.98	
Sample 17	Case 3	Male	86		Frontal cortex	4.30	1.63	MV
Sample 18					Hippocampus	6.45	1.43	
Sample 19	Case 4	Female	60		Frontal cortex	5.61	1.25	MM
Sample 20					Hippocampus	5.10	1.04	
Sample 21	Case 5	Female	72		Frontal cortex	4.46	1.58	MM
Sample 22					Hippocampus	5.92	1.22	
Sample 23	Case 6	Female	54		Frontal cortex	5.91	0.90	MV
Sample 24					Hippocampus	5.56	0.83	

**Table 3:** Detailed information on the primer sets used in this study.

Purpose		Name	Sequence	Size	Annealing Temperature
Sanger sequencing	Amplification	F	CAACCGCTACCCACCTCAG	564 bp	60 °C
		R	AGGACCATGCTCGATCCTCT		
Pyrosequencing	Amplification	PF	AGGCCCATGGATGAGTACAG	161 bp	60 °C
		Biotin_PR	TGGGTGATACACATCTGCTCAAC		
	Sequencing	Seq	AGGGGGAGAACTTCA		

## Data Availability

All data are available from the corresponding authors upon reasonable request.
